# Transaneurysmal Repair of a Giant Calcified Submitral Left Ventricular Aneurysm

**DOI:** 10.21470/1678-9741-2019-0113

**Published:** 2020

**Authors:** Sachin Sanagar, Shantesh Kaushik, Sanjeev Jadhav, Saurabh Tiwari, Rahul Gupta

**Affiliations:** 1 Department of Cardiothoracic Surgery, Apollo Hospitals, Navi Mumbai, India.; 2 Department of Cardiac Anesthesia, Apollo Hospitals, Navi Mumbai, India.; 3 Department of Cardiology, Apollo Hospitals, Navi Mumbai, India.

**Keywords:** Submitral Aneurysm, Calcified Aneurysm, Cardiac, Mitral Valve

## Abstract

Submitral left ventricular aneurysm is a rare cardiac pathology with very few cases reported in the literature. These are nonischemic aneurysms mostly reported from Africa. Patients with submitral aneurysm exhibit varied clinical manifestations. We report a case of calcified submitral aneurysm and its successful surgical management through a transaneurysmal approach.

**Table:** 

Abbreviations, acronyms & symbols	
ECG	= Electrocardiogram
SMA	= Submitral aneurysm

## INTRODUCTION

Submitral aneurysms have been described predominantly in African populations, although they have also been reported in mixed races and Caucasians^[1]^. Recent studies have revealed a congenital base of the submitral aneurysm as complex embryogenesis, causing weakness in the left submitral ventricular wall, although a genetic link has been suspected because of the racial predilection. The other suggested etiologies are infection and inflammation, such as tuberculosis, rheumatic endocarditis and Takayasu’s arteritis^[2]^. Of the different possible surgical approaches, we successfully used a transaneurysmal approach.

## CASE REPORT

**Fig. 1:**
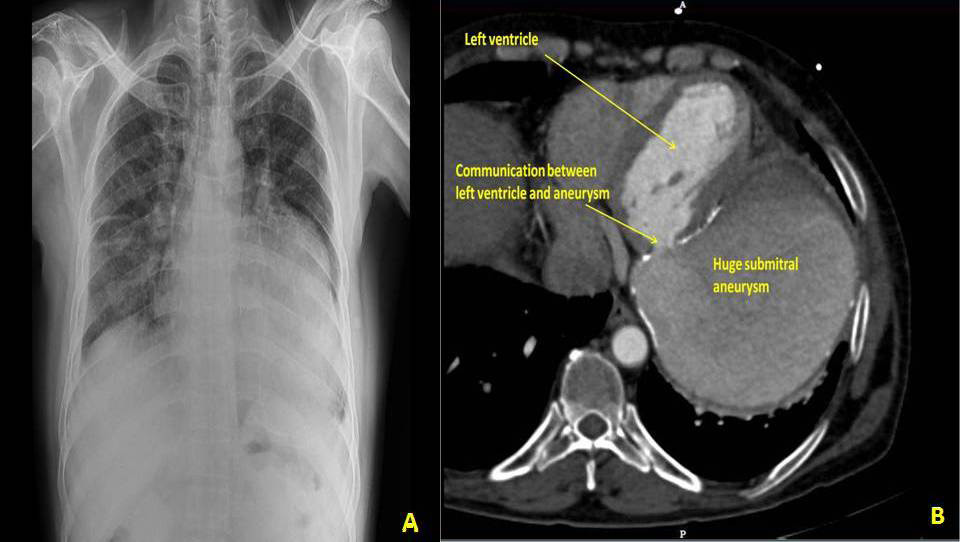
(A) Chest roentgenogram showing calcified cystic lesion in the left hilar region; (B) Computed tomography showing communication between left ventricle and submitral aneurysm.

**Fig. 2:**
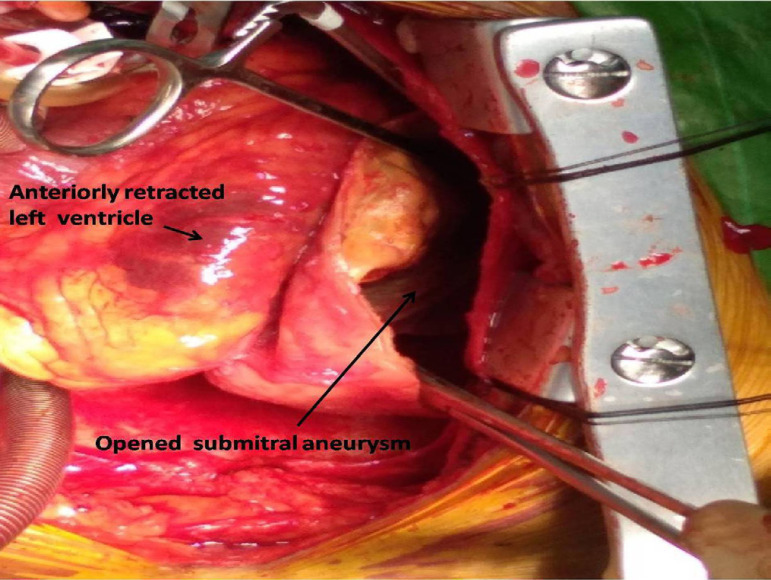
Opened submitral aneurysm.

A 53-year-old schizophrenic male presented with progressive worsening of dyspnea on exertion in the last 3 years, with worsening for 2 weeks. He was treated for heart failure 3 months earlier at another hospital. There was no history of trauma, rheumatic fever or tuberculosis. At clinical examination, heart rate was 96 bpm and blood pressure was 112/74 mmHg. The apex was in the 6^th^ left intercostal space, 3 cm lateral to the midclavicular line. On auscultation, a mild S1 and a late apical systolic murmur grade 3/6 were present. The electrocardiogram (ECG) showed sinus rhythm. Chest roentgenogram showed calcified cystic lesion in the left hilar region (Figure 1A). Transthoracic echocardiography revealed a large submitral aneurysm of size 11×8×7 cm posterolateral to the left ventricle with a 1.2 cm neck. Left ventricular ejection fraction was 50%, with mild mitral regurgitation without pulmonary hypertension. Cardiac computed tomography revealed an aneurysm size of 11.4×11.1 cm, occupying the inferior left paracardiac hemithorax (Figure 1B). The ECG-gated cardiac angiogram showed opacification with contrast through the small neck inferolateral to the mitral valve. The aneurysm wall was 3-5 mm thick with curvilinear calcification. Coronary angiography showed normal coronary arteries. Left ventriculography confirmed the contrast flow from the left ventricle to the aneurysm. The aneurysm was just abutting the obtuse marginal branch of the left circumflex coronary artery. Open repair of the aneurysm through a midline sternotomy was planned. Cardiopulmonary bypass with aortobicaval cannulation and cardioplegic arrest allowed dissection of the aneurysm. The left ventricular aneurysm arising from the posterior submitral region was opened and its contents evacuated (Figure 2). The free aneurysmal calcified wall was partially excised, keeping the lateral part adherent to the hilum, *in situ*. On inspection, mitral subvalvar apparatus was intact. The defect was repaired with a bovine pericardial patch using the 2-0 Prolene continuous suture technique. Free aneurysmal wall margins were sutured over the pericardial patch. Intraoperative transesophageal echocardiography confirmed complete repair without leakage. The patient was extubated after 10 hours. Postoperatively, his schizophrenic symptoms worsened, for which he received treatment and responded well, and was discharged on the 11^th^ postoperative day. Histopathological examination of the aneurysmal wall showed bands of dense eosinophilic tissue, areas of fibroid necrosis, hemorrhage and foci of calcification.

Doppler echocardiography after one month showed good left ventricular function with mild mitral regurgitation.

## DISCUSSION

Most submitral aneurysms (SMAs) are reported in Africa, with the first case reported in 1962 by Abrahams et al.^[3]^. Recently, few cases have been reported in the Indian subcontinent^[4]^. Clinically, submitral aneurysms are characterized by heart failure, mitral insufficiency, and absence of coronary disease associated with thromboembolic cardiomyopathy and cardiac arrhythmias^[5]^. Compression of coronary arteries by the aneurysm can have ischemic manifestations. Transthoracic Doppler is usually diagnostic. Aneurysm details such as neck size and extension can be accurately defined with the help of magnetic resonance imaging. Contrast-enhanced computed tomography angiography reveals the SMA calcification and compression of the adjacent structures. The aneurysm neck closure with bovine pericardium is the treatment of choice. Either sternotomy or left thoracotomy approach can be used. Aneurysm neck can be accessed through transmitral, left atrial or transaneurysmal route^[4,6]^. Closing the aneurysm wall over the patch ensured good hemostasis and reduced the chances of patch aneurysm formation.

## CONCLUSION

Submitral left ventricular aneurysm is a rare entity. In our case, transaneurysmal approach was adopted, as the need for mitral intervention was unlikely. Definitive diagnosis with thorough preoperative evaluation and good surgical technique lead to successful surgical outcomes for submitral aneurysms.

**Table:** 

Author's roles & responsibilities	
SS	Substantial contributions to the conception or design of the work; or the acquisition, analysis, or interpretation of data for the work; drafting the work or revising it critically for important intellectual content; final approval of the version to be published
SK	Substantial contributions to the conception or design of the work; or the acquisition, analysis, or interpretation of data for the work; drafting the work or revising it critically for important intellectual content; final approval of the version to be published
SJ	Substantial contributions to the conception or design of the work; or the acquisition, analysis, or interpretation of data for the work; final approval of the version to be published
ST	Substantial contributions to the conception or design of the work; or the acquisition, analysis, or interpretation of data for the work; final approval of the version to be published
RG	Substantial contributions to the conception or design of the work; or the acquisition, analysis, or interpretation of data for the work; final approval of the version to be published
